# Identification and Analysis of Potential Key Genes Associated With Hepatocellular Carcinoma Based on Integrated Bioinformatics Methods

**DOI:** 10.3389/fgene.2021.571231

**Published:** 2021-03-09

**Authors:** Zhuolin Li, Yao Lin, Bizhen Cheng, Qiaoxin Zhang, Yingmu Cai

**Affiliations:** ^1^Department of Clinical Laboratory, The First Affiliated Hospital of Shantou University Medical College, Shantou, China; ^2^Department of Plastic Surgery and Burn Center, The Second Affiliated Hospital of Shantou University Medical College, Shantou, China

**Keywords:** hepatocellular carcinoma, bioinformatics, differentially expressed genes, survival, biomarker, GEO, TCGA

## Abstract

**Background:**

Hepatocellular carcinoma (HCC) is a type of primary liver tumor with poor prognosis and high mortality, and its molecular mechanism remains incompletely understood. This study aimed to use bioinformatics technology to identify differentially expressed genes (DEGs) in HCC pathogenesis, hoping to identify novel biomarkers or potential therapeutic targets for HCC research.

**Methods:**

The bioinformatics analysis of our research mostly involved the following two datasets: Gene Expression Omnibus (GEO) and The Cancer Genome Atlas (TCGA). First, we screened DEGs based on the R packages (limma and edgeR). Using the DAVID database, the Gene Ontology (GO) and Kyoto Encyclopedia of Genes and Genomes (KEGG) enrichment analyses of DEGs were carried out. Next, the protein-protein interaction (PPI) network of the DEGs was built in the STRING database. Then, hub genes were screened through the cytoHubba plug-in, followed by verification using the GEPIA and Oncomine databases. We demonstrated differences in levels of the protein in hub genes using the Human Protein Atlas (HPA) database. Finally, the hub genes prognostic values were analyzed by the GEPIA database. Additionally, using the Comparative Toxicogenomics Database (CTD), we constructed the drug-gene interaction network.

**Results:**

We ended up with 763 DEGs, including 247 upregulated and 516 downregulated DEGs, that were mainly enriched in the epoxygenase P450 pathway, oxidation-reduction process, and metabolism-related pathways. Through the constructed PPI network, it can be concluded that the P53 signaling pathway and the cell cycle are the most obvious in module analysis. From the PPI, we filtered out eight hub genes, and these genes were significantly upregulated in HCC samples, findings consistent with the expression validation results. Additionally, survival analysis showed that high level gene expression of CDC20, CDK1, MAD2L1, BUB1, BUB1B, CCNB1, and CCNA2 were connected with the poor overall survival of HCC patients. Toxicogenomics analysis showed that only topotecan, oxaliplatin, and azathioprine could reduce the gene expression levels of all seven hub genes.

**Conclusion:**

The present study screened out the key genes and pathways that were related to HCC pathogenesis, which could provide new insight for the future molecularly targeted therapy and prognosis evaluation of HCC.

## Introduction

Accounting for 75-85% of all primary liver cancer, hepatocellular carcinoma (HCC) is the main histological classification of liver cancer, which is the fourth most frequent cause of cancer-related death globally (Harris et al., 2019; Yang J.D. et al., 2019). The liver is the second most common cancer-prone organ, after the lungs, as was shown by the recent cancer study in China (Fu and Wang, 2018). On the whole, the estimated morbidity of HCC per 100,000 world standard population is 40.0 in males and 15.3 in females (Zhu et al., 2016). Major risk factors for HCC include genetic predisposition, epigenetic variation, chronic hepatitis B infection, hepatitis C virus infection, smoking, obesity, aflatoxin exposure, and diabetes (Puszyk et al., 2013; Baecker et al., 2018). Transplantation is the most useful way to treat HCC; however, after the transplantation process, the tumor recurrence and metastasis rates are high (Au and Chok, 2018; Aufhauser et al., 2018). More than 70% of patients at advanced stage are not suitable for transplantation, whether due to the tumor burden or liver dysfunction (Wang et al., 2019). Therefore, it is urgent to recognize new biomarkers that can act as molecular targets for therapy, and predictors of the prognosis of HCC. With the development of times and technological progress, microarray and high-throughput sequencing technologies have matured and become more reliable, and public databases are improving, such as the Gene Expression Omnibus (GEO)^1^ and the Cancer Genome Atlas (TCGA)^2^. The advancement of microarray (Yang X. et al., 2018) and high throughput sequencing technologies (Weinstein et al., 2013) has provided a highly efficient tools to explore key genetic or epigenetic changes in disease to identify biological markers that can be applied to disease diagnosis, therapy, and prognosis (Weinstein et al., 2013; Wang et al., 2018; Yang X. et al., 2018; Li et al., 2019). Additionally, the application of integrated bioinformatics methods in cancer research can solve the problem of different results due to errors caused by different technical platforms or small sample size, thus finding much valuable biological information (Liu X. et al., 2018; Deng et al., 2019; Yan et al., 2019; Yang K. et al., 2019).

In this research, by analyzing and distinguishing genes in human HCC samples and normal hepatocyte samples using TCGA and GEO datasets, we firstly screened out differentially expressed genes (DEGs). Then, GO and KEGG pathway enrichment analyses were applied in the further exploration of the main biological functions, which regulated by the DEGs. After that, the final step is to utilize a protein–protein interaction (PPI) network, survival analyses and drug-gene interaction network analyses to ascertain crucial genes and pathways which affecting the pathogenic mechanism and prognosis of HCC patients.

## Materials and Methods

### Gene Expression Datasets

The microarray gene expression dataset of GSE121248, which comprises 70 hepatocellular carcinoma samples and 37 normal liver samples, was obtained from the GEO website and exploited as discovery dataset to identify DEGs. The included dataset met the following criteria: (1) dataset included human HCC samples and normal liver samples. (2) they contained at least ten samples. (3) dataset was obtained from the Affymetrix Human Genome U133 Plus 2.0 Array [HG-U133_Plus_2] microarray platform. The raw RNA sequencing data, which comprises 374 HCC samples and 50 normal liver tissue samples, was selected from the TCGA liver hepatocellular carcinoma (TCGA-LIHC) dataset and used as a validation dataset.

### Identification of DEGs

We used the R language to analyze the original CEL files of the GSE121248 dataset.

The preprocessing procedures: using the “affy” R package to RMA background correction, Log2conversion, Quantile normalization, and Median polish algorithm summarization (Bolstad et al., 2003; Gautier et al., 2004). Using the bioconductor annotation package to convert microarray data probes into gene symbol. If multiple probes were mapped to a gene symbol, take the average value as the final expression value of the gene (Zhang et al., 2018). Next, | log2fold change (FC)| > 1 and adjusted *p* value <0.05 were used to select the DEGs between tumor and normal tissues using the LIMMA package (Ritchie et al., 2015; Nagy et al., 2018).

### DEGs Validation Using the TCGA Dataset

The DEGs from the GSE121248 dataset were validated using the TCGA-LIHC dataset.

The edgeR package of R software was applied to normalize and analyze the TCGA-LIHC dataset (Robinson et al., 2010). | log2fold change (FC)| > 1 and *p*-value <0.05 were considered significant differences. The overlapping DEGs between GSE121248 and TCGA-LIHC datasets were clustered using the pheatmap and were retained for further study. The overlapping DEGs were analyzed using VennDiagram and ggplot2 packages in R software to draw Venn diagrams and volcano plots, to visualize the identified DEGs (Chen and Boutros, 2011).

### Functional Enrichment Analysis of Overlapping DEGs

We used the Database for Annotation, Visualization and Integrated Discovery (DAVID version 6.8)^3^ to elucidate potential GO function [including biological processes (BP), molecular functions (MF), cellular components (CC)] and signaling pathways (KEGG) related to the overlapping DEGs (Dennis et al., 2003; Kanehisa et al., 2017). We used threshold *p*-value 0.05.

### Protein–Protein Interaction Network Construction and Module Analysis

The Search Tool for the Retrieval of Interacting Genes (STRING version 11)^4^ database was one of the largest online databases of known protein-protein interactions covering the largest number of species (Szklarczyk et al., 2017). The parameter of interactions was set with a confidence score >0.7. The confidence score refers to the strength of data support in terms of the thickness of the line. Confidence score >0.7 means high confidence. Overlapping DEGs were entered into Cytoscape software (version 3.7.2)^5^ to construct and analyze PPI network (Shannon et al., 2003). Moreover, the Cytoscape plug-in MCODE was used to screen crucial clustering modules in the entire network (Bader and Hogue, 2003).

### Identification of Hub Genes

The Cytoscape plug-in CytoHubba was used to calculate the protein node degree (Chin et al., 2014; Cao et al., 2018). The top three methods [(Maximal Clique Centrality (MCC), Maximum Neighborhood Component (MNC), and Density of Maximum Neighborhood Component (DMNC)] were selected to provide the analyzed results. Each method displayed their top ten genes. A Venn diagram was generated to visualize common hub genes based on these three methods.

### Expression Analysis of Hub Genes in Multiple Databases

The hub genes mRNA expression levels were finally validated in two databases, Gene Expression Profiling Interactive Analysis (GEPIA)^6^ (Tang et al., 2017) and Oncomine. Oncomine (Version4.5)^7^ is an online database that has the comprehensive cancer mutation spectrum, gene expression data and related clinical information, which can be used to discover new biomarkers or new therapeutic targets (Rhodes et al., 2004). In addition to detecting the mRNA expression levels of the hub genes, we also investigated the protein levels in HCC tissues and normal liver tissues using the human protein atlas database (HPA v19)^8^ (Thul and Lindskog, 2018).

### Survival Analysis

Gene Expression Profiling Interactive Analysis is a newly developed online database for cancer and normal gene expression profiling. In the current study, the overall survival of each hub gene was analyzed using LIHC dataset in the GEPIA database. The patients were divided into two groups (the high- and low-expression group) according to the median expression level of each hub gene. This division method could evaluate the difference in overall survival probability between these two groups. We were drawn the overall survival curves of each hub gene using the GEPIA database, with a *p*-value <0.05.

### Drug-Gene Interaction Network Analysis

The Comparative Toxicogenomics Database (CTD)^9^, an online database providing information on the interactions between gene products and chemotherapeutic drugs, and their relationships to diseases) was used to construct the chemotherapeutic drug-gene interaction network (Davis et al., 2019). The networks were visualized by Cytoscape software 3.7.2^10^.

## Results

### Identification of DEGs

**FIGURE 1:**
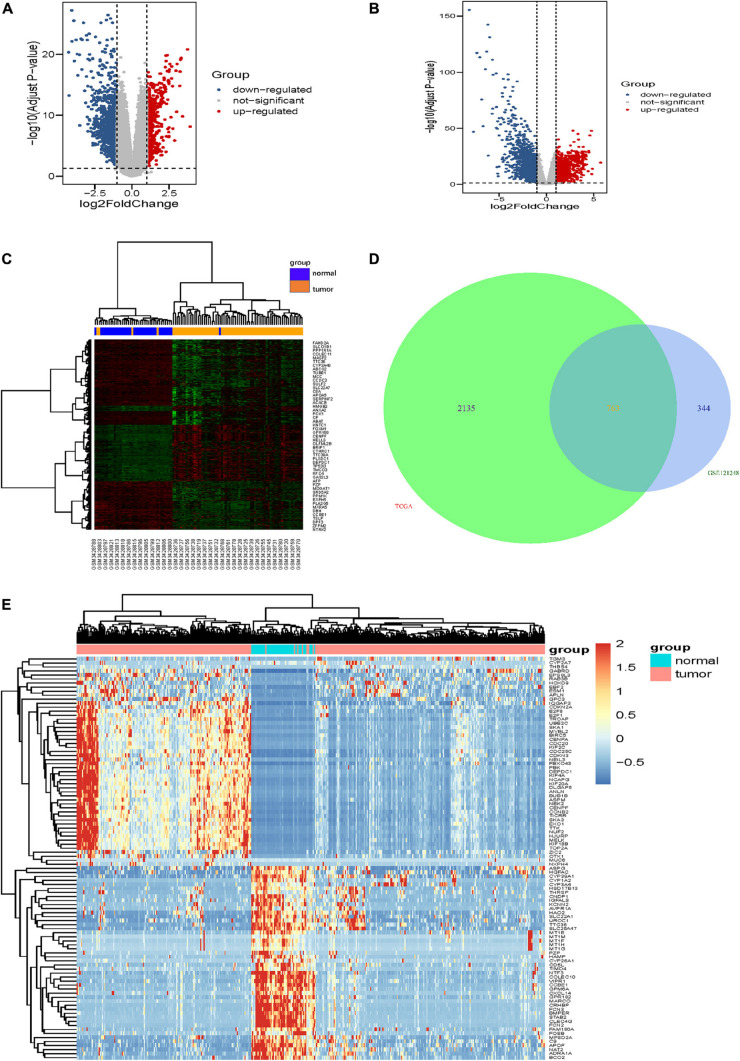
Identification of DEGs. **(A,B)** show the volcano maps of DEGs for **(A)** GSE121248 dataset, **(B)** TCGA-LIHC dataset. **(C)** The heatmap of the top 50 DEGs in dataset GSE121248. The green color and red color in the heatmap indicate low and high expression of DEGs. **(D)** Venn diagrams of the DEGs between the GSE121248 dataset and the TCGA-LIHC dataset. **(E)** The heatmap of the top 100 overlapping DEGs according to the value of | logFC| in TCGA-LIHC dataset. The color in heatmaps from green to red shows the progression from down-regulation to up-regulation.

The gene expression dataset of GSE121248, which contains 70 LIHC samples and 37 normal liver samples, was analyzed in the limma package using | logFC| > 1 and corrected *p*-value <0.05 of R software. In total, 1,518 DEGs (557 high expression genes and 961 low expression genes) were identified between HCC tissue samples and normal liver tissue samples. The volcano map and heatmap of all DEGs are shown in Figures 1A,C. Additionally, compared with normal liver tissues in the TCGA-LIHC dataset, 2,898 DEGs were obtained in LIHC tissues, comprising 1,299 upregulated genes and 1,599 downregulated genes (Figure 1B). Furthermore, 763 overlapping DEGs (247 high expression genes and 516 low expression genes) were identified between the GSE121248 and TCGA-LIHC datasets using a Venn diagram (Figure 1D). Figure 1E shows clustering analysis results of the 763 overlapping DEGs based on the TCGA-LIHC dataset.

### Enrichment Analysis of Overlapping DEGs

**FIGURE 2:**
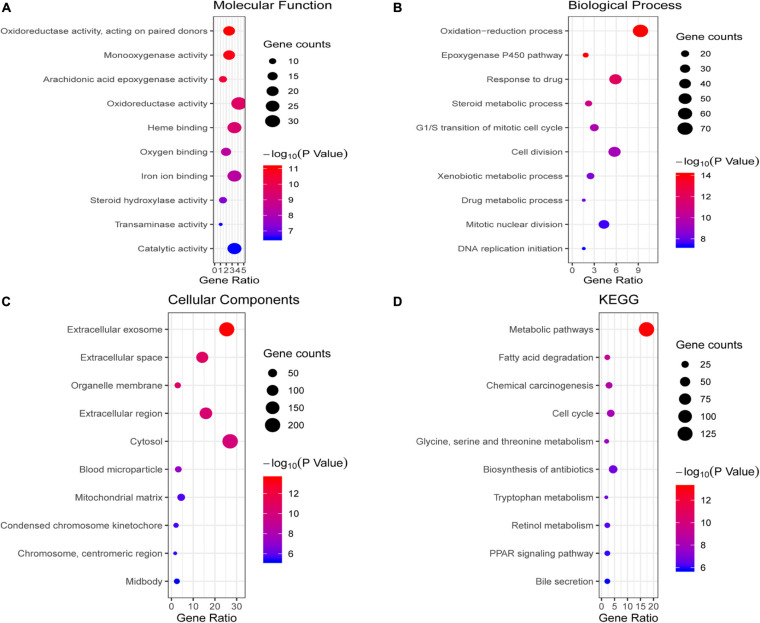
Enrichment analysis of the overlapping DEGs. **(A–C)** illustrate the GO enrichment analysis results: **(A)** molecular function, **(B)** biological process and **(C)** cellular components. **(D)** KEGG pathway enrichment analysis results.

We conducted GO and KEGG pathway enrichment analysis to further elucidate potential biological functions associated with the 763 overlapping DEGs of HCC. The GO analysis results of the DEGs were classified into molecular functions, biological processes and cellular components. For molecular functions, the overlapping DEGs were mainly associated with oxidoreductase activity, monooxygenase activity, heme binding and oxygen binding (Figure 2A). In the BP category, the epoxygenase P450 pathway, oxidation-reduction process, response to drug and cell division were enriched (Figure 2B). In the CC category, they were enriched in extracellular regions, such as extracellular exosomes and the extracellular space (Figure 2C). The pathway enrichment analysis results showed that overlapping DEGs mainly participated in multiple metabolism pathways, such as fatty acid degradation, glycine, serine and threonine metabolism, and tryptophan metabolism (Figure 2D).

### PPI Network Establishment and Module Analysis

**FIGURE 3:**
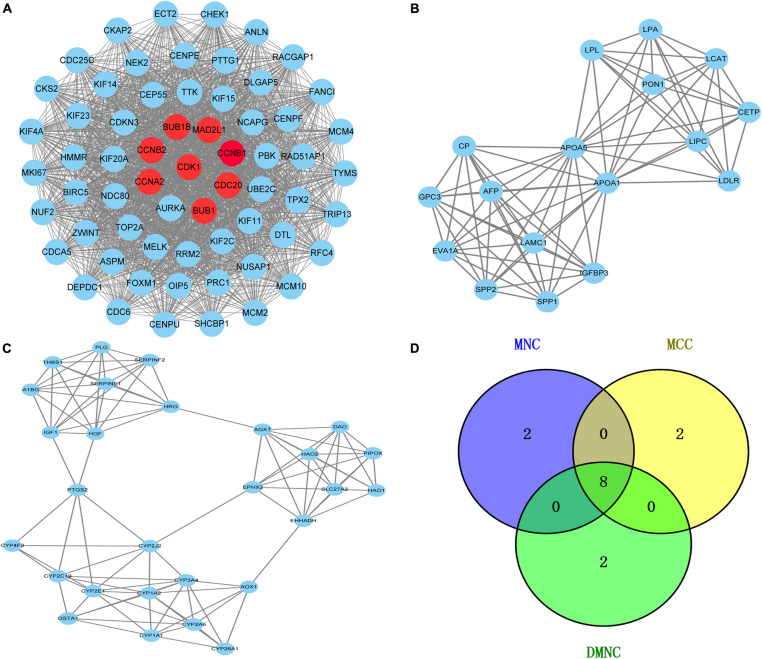
Venn diagram and the top three clustering modules of PPI network. **(A)** Module 1 with an MCODE score of 56.5. The red nodes are the hub genes. **(B)** Module 2 obtained a score of 10.0 from MCODE. **(C)** Module 3 with an MCODE score of 7.4. Edges represent the protein-protein associations. The higher the module score, the more important the module is in the PPI network. **(D)** Venn diagrams of the hub genes between three methods (MNC, MCC, and DMNC).

To further reveal the potential relationships between proteins encoded by DEGs, a PPI network was constructed using the STRING database. Network analysis of overlapping DEGs revealed 526 nodes and 4,173 edges in the PPI network. Additionally, we conducted module analysis using the MCODE plug-in to detect crucial clustering modules. In total, 29 clusters were obtained in MCODE, and the top three modules with the highest scores were selected as hub modules. Module 1 contained 63 nodes and 1,752 edges with the highest score of 56.516 and was mainly enriched in cell cycle, oocyte meiosis, P53 signaling pathway and progesterone-mediated oocyte maturation (Figure 3A). Module 2 contained 17 nodes and 80 edges with a score of 10 and mainly participated in PPAR signaling pathway and glycerolipid metabolism (Figure 3B). Module 3 comprised 28 nodes and 100 edges with a score of 7.407 and was mainly implicated in Chemical carcinogenesis, Peroxisome, Metabolic pathways and Drug metabolism cytochrome P450 (Figure 3C).

### Hub Genes Selection From the PPI Network

The Cytoscape plug-in cytoHubba including the top three algorithms (MCC, MNC, and DMNC) was applied to select hub genes, and the top 10 genes were selected by each of the three methods. The Venn diagram identified eight overlapping hub genes based on these three methods (Figure 3D): cell division cycle protein 20 homolog (CDC20), cyclin-dependent kinase1 (CDK1), mitotic spindle assembly checkpoint protein MAD2A (MAD2L1), threonine-protein kinase BUB1 (BUB1), threonine-protein kinase BUB1 beta (BUB1B), mitotic-specific cyclin-B1 (CCNB1), mitotic-specific cyclin-B2 (CCNB2) and cyclin-A2 (CCNA2). These eight hub genes were used for further analysis.

### Validation of Hub Genes in Multiple Databases

**FIGURE 4:**
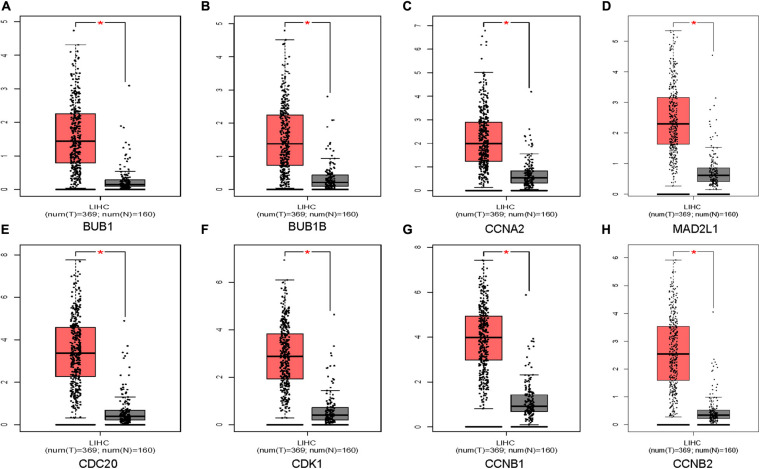
Validation of eight hub genes mRNA expression levels in HCC tissues vs. normal liver tissues using the GEPIA database **(A–H)**. The red color represents the tumor samples and the gray color represents the normal liver samples.

**FIGURE 5:**
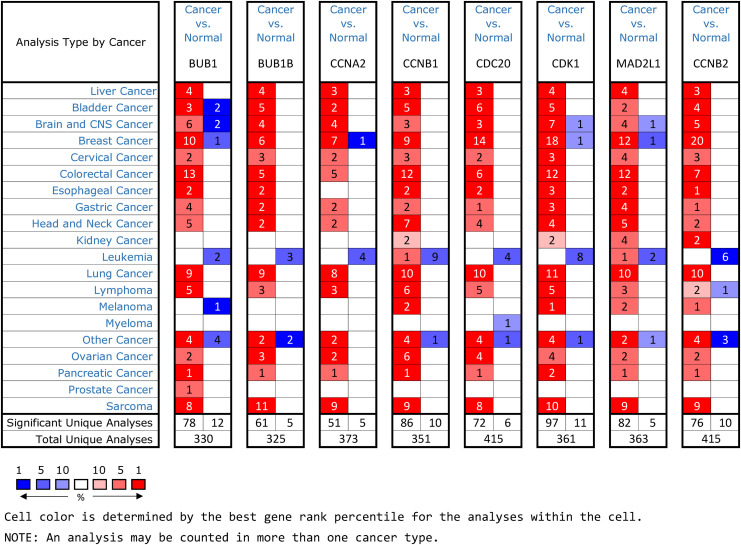
An summary of mRNA expression results of 8 hub genes in multiple tumors using the Oncomine database. The numbers in colored cells show the quantities of datasets with high (red) or low (blue) mRNA expression of the hub genes.

**FIGURE 6:**
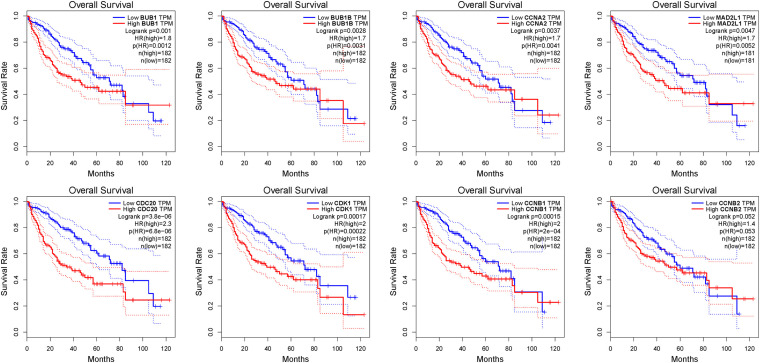
The OS analysis of 8 hub genes in the HCC patients using the GEPIA database. The red curve is the high expression group and the blue curve is the low-expression group. *p*-value < 0.05.

**FIGURE 7:**
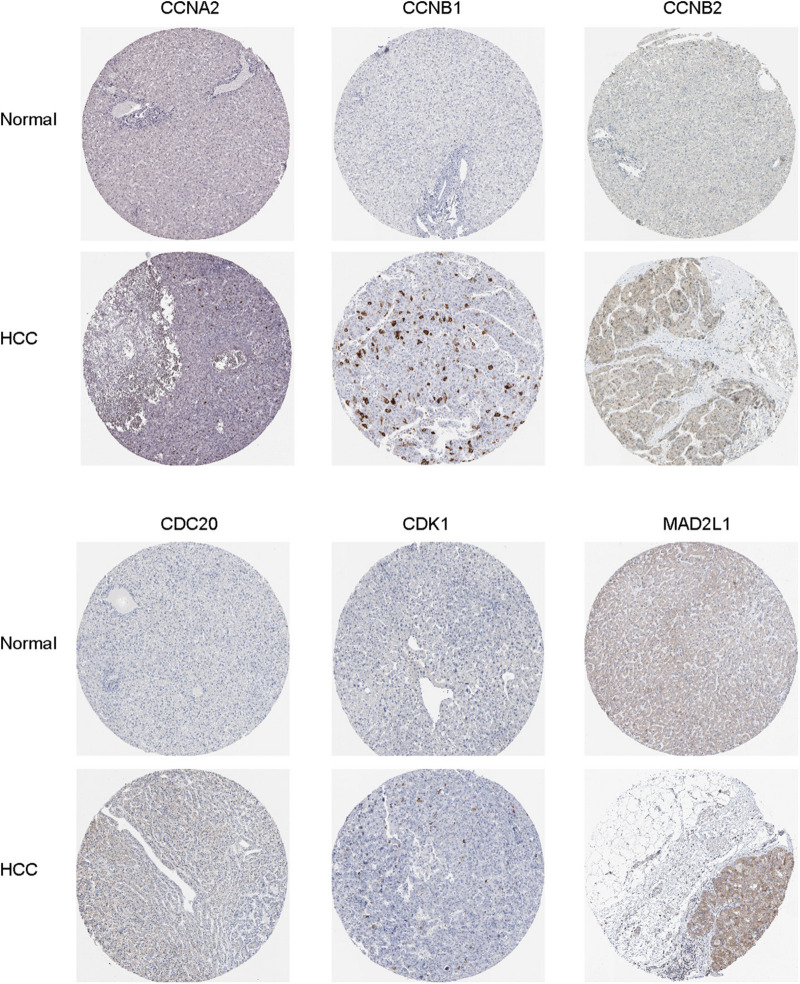
Immunohistochemical staining analysis of hub genes (CCNA2, CCNB1, CCNB2, CDC20, CDK1, and MAD2L1) in HCC tissues and normal liver tissues.

Oncomine and GEPIA were applied to validate the differentially expression levels of 8 hub genes between HCC tissues and normal liver tissues in HCC. These eight hub genes were all remarkably overexpressed in HCC samples (Figure 4). Moreover, a summary of hub genes in multiple tumors indicated that hub genes were significantly overexpressed in HCC (Figure 5). Furthermore, we also investigated the protein expression levels in HCC tissue samples and normal liver tissue samples using the human protein atlas database. Because the HPA dataset could not provide immunohistochemical information on BUB1 and BUB1B, we showed the results of the remaining six staining pairs in Figure 6. The protein expression levels of hub genes were agreed with the mRNA expression results, and most genes were overexpressed in HCC tissue (Figure 7). These findings indicate that the overexpression of these hub genes may play a critical role in HCC mechanism.

### Survival Analysis

We further used the GEPIA database to analyze the prognostic value of these 8 hub genes in HCC patients. The survival analysis of patients in the GEPIA database was based on the TCGA-LIHC data set. We used threshold *p*-value 0.05 and calculated the hazards ratio based on Cox PH Model (Xu et al., 2020). The relatively higher expression of CDC20 (HR = 2.3; P = 3.8e-06), CDK1 (HR = 2; *P* = 0.00017), MAD2L1 (HR = 1.7; *P* = 0.0047), BUB1 (HR = 1.8; *P* = 0.001), BUBIB (HR = 1.7; *P* = 0.0028), CCNB1 (HR = 2; *P* = 0.00015), and CCNA2 (HR = 1.7; *P* = 0.0037) were associated with a poor prognosis in HCC patients, while only CCNB2 (HR = 1.4; *P* = 0.052) showed no statistical significance in the overall survival of patients (Figure 6).

### Drug-Gene Interaction Network Analysis

**FIGURE 8:**
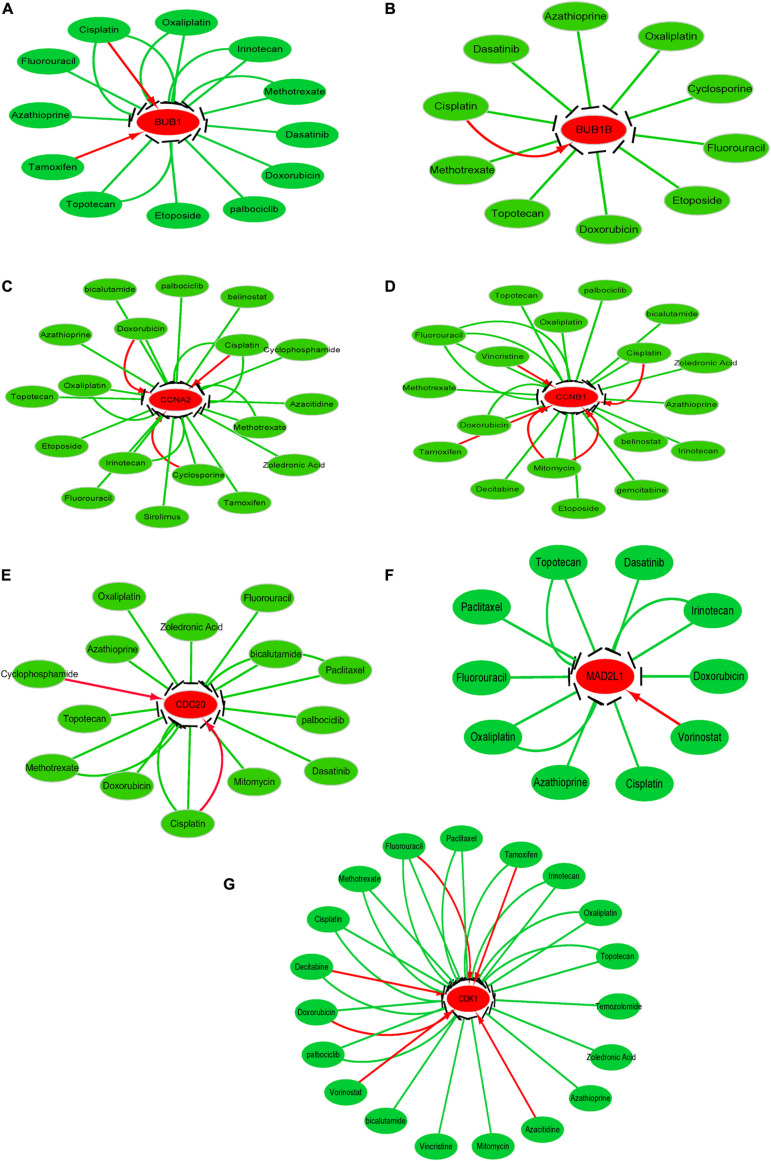
Drug-gene interactions network with chemotherapeutic drugs and seven hub genes was constructed using the CTD database. **(A–G)** shows the relationship between existing chemotherapeutic drugs and the expression levels of hub genes. **(A)** BUB1, **(B)** BUB1B, **(C)** CCNA2, **(D)** CCNB1, **(E)** CDC20, **(F)** MAD2L1, and **(G)** CDK1. The red and green arrows represent that the chemotherapy drugs will increase or decrease the expression of the hub genes. The number of arrows between hub genes and chemotherapy drugs indicates the number of references supported by previous studies.

To investigate the potential information on the interactions between hub genes and cancer chemotherapeutics drugs, we used the CTD database to construct chemotherapeutics drug-gene interaction network. Various drugs could influence the mRNA expression level of seven hub genes, namely, CDC20, CDK1, MAD2L1, CCNA2, CCNB1, BUB1, and BUB1B (Figure 8). However, only topotecan, oxaliplatin and azathioprine could reduce expression levels of all seven hub genes.

## Discussion

Hepatocellular carcinoma is a type of primary liver tumor with poor prognosis and high mortality, and the progress in its diagnosis and treatment has always attracted widespread attention from researchers around the world. Because the high recurrence and metastasis rate of HCC remains a challenge, identifying new molecules as biological markers is urgently needed. Integrated bioinformatics analysis, which focuses on screening of DEGs, discovering hub node of network-based and doing survival analysis, which has been diffusely used to recognize latent biological markers related to cancer diagnosis, therapy, and prognosis estimation. In recent years, increasing researches have demonstrated that abnormal gene expression is a factor in the tumorigenesis and development, so it is feasible to screen differential genes as biomarkers to assist diagnosis and treatment. In 2017, by developing an integrated approach including GO and KEGG analysis, PPI network creation, hub gene identification, and overall survival analysis, Li L. et al. (2017) picked out 16 hub genes for HCC from three GEO datasets, five of which may be playing a part in the occurrence, development, invasion, metastasis or recurrence of HCC. In 2018, Zhang L. et al. (2018) used bioinformatics methods to select 10 genes from the GEO dataset GSE64041 for the identification of hub genes and pathways of HCC. Gu et al. (2020) recognized 13 crucial genes correlated with progression and prognosis of HCC from the TCGA-LIHC dataset by weighted gene coexpression network analysis. Compared with previous similar studies, our study not only integrated a large sample size of mRNA expression data from the GEO database but also analyzed RNA sequencing result and clinical data from the TCGA-LIHC database to screen out potential hub genes in HCC. And in the second place, this study validated the DEGs through multiple databases. Finally, we explored the relationship between seven hub genes and existing drugs for cancer therapy, which may provide some guidance for the molecular targeting therapy of HCC in the future.

In our research, DEGs in HCC based on the GEO expression profile of GSE121248 (70 HCC samples and 37 normal samples) and TCGA-LIHC RNA sequencing data (374 HCC samples and 50 normal samples) were identified by bioinformatics analysis. In total, 763 significantly robust DEGs, including 247 upregulated DEGs and 516 downregulated DEGs, were identified. The enrichment analysis results of GO indicated that the DEGs were mostly relevant to “oxidoreductase activity, acting on paired donors,” “monooxygenase activity,” “arachidonic acid epoxygenase activity,” “oxidation-reduction process,” “epoxygenase P450 pathway,” “response to drug,” “extracellular exosome,” “extracellular region,” and “cytosol.” The analysis of KEGG pathway showed that the DEGs were mainly concentrated in the following: “fatty acid degradation pathway,” “metabolic pathways,” “chemical carcinogenesis pathway,” “cell cycle pathway,” and “biosynthesis of antibiotics pathway.” Previous studies have reported that the arachidonic acid-derived metabolites and cytochrome P450 epoxygenase CYP2J2 possibly play vital roles in regulating malignant tumor, stimulating tumor cell growth, and inhibiting tumor cell apoptosis (Liu L. et al., 2011; Xu et al., 2011; Yarla et al., 2016). Additionally, metabolic pathways are important for cancer cell survival because the metabolic demands of cancer cells are often expressed as increased, and HCC shows a significant alteration in lipid metabolism (Pope et al., 2019). Moreover, dysregulation of the cell cycle processes and mitotic cell cycle plays a vital role in the tumorigenesis and progression (Williams and Stoeber, 2012; Wlodarchak and Xing, 2016). These theories are consistent with our results in GO and KEGG enrichment analysis.

Through building PPI network and analyzing it, we identified crucial hub genes in the PPI network, including CDC20, CDK1, MAD2L1, BUB1, BUB1B CCNB1, CCNB2, and CCNA2. Using Oncomine and GEPIA validation, the mRNA expression of these eight hub genes in HCC samples was higher than normal liver samples, the finding that was in accord with the microarray results. Subsequently, HPA database data displayed that the protein and mRNA expression of hub genes were consistent, and most genes were overexpressed in HCC tissue. To inquire prognostic biological markers of HCC, we applied the GEPIA to analyze the influence of hub genes expression level on survival of HCC patients and found that, except CCNB2, the high level gene expression of CDC20, CDK1, MAD2L1, BUB1, BUB1B, CCNB1, and CCNA2 were related to HCC patients poor overall survival. Therefore, these seven genes may be functional in HCC occurrence and development.

It was reported that high expression of CDC20 (cell division cycle protein 20) is associated with poor survival in astrocytoma (Ding et al., 2017), cutaneous squamous cell carcinoma (Chu et al., 2019) and pancreatic ductal adenocarcinoma (Dong et al., 2019). CDC20 promotes the progression of prostate cancer by stabilizing hypo-catenin in tumor-like dry cells (Zhang et al., 2019). However, the expression of cell division cycle protein 20 in HCC still lacks accurate experimental data. As a part of the Ser/Thr protein kinase family, CDK1 (cyclin-dependent kinase 1) is a key molecule that controls the eukaryotic cell cycle. By phosphorylating Bora, Cyclin A/cdk1 could facilitate the phosphorylation, activation and mitotic entry of Aurora A-dependent Plk1 (Vigneron et al., 2018). It is reported that CDK1 overexpression has been found in colorectal cancer, pancreatic ductal adenocarcinoma and thyroid cancer (Zhang P. et al., 2018; Piao et al., 2019; Zheng et al., 2019). It was also reported that CDK1 amplification rate in HCC tissues was usually up to 46% (18/39), which was meaningfully related to poor overall survival (*p* = 0.008) (Wu et al., 2018). These results were in accord with our study findings.

As a pro-oncogene upregulated in gastric cancer, MAD2L1 (mitotic arrest deficient 2-like protein 1) can be downregulated expression by miR-30a-3p, resulting in inhibition of the proliferation of gastric cancer cells (Wang et al., 2019). Besides, by restraining MAD2L1, miR-200c-5p can inhibit HCC cells proliferation, migration and invasion (Li Y. et al., 2017), suggesting that MAD2L1 can be used in HCC patients prognostic evaluation and targeted therapy. As a cyclin controlling the G1/S and G2/M phases in the cell cycle, CCNA2 (cyclin-A2) is more expressed in CRC samples than in normal samples. The reduction of CCNA2 gene expression would disrupt cell cycle progression and induce apoptosis, thus significantly inhibiting the growth of CRC cells (Gan et al., 2018). By maintaining the expression of CCNA2 protein and the production of arginine, arginine metabolic enzyme argininosuccinate lyase (ASL) can promote the production of nitric oxide synthase, thus promoting the formation of HCC (Hung et al., 2017).

As a mitotic checkpoint serine/threonine kinase, BUB1 is related to tumorigenesis in many cancers. shRNA silencing inhibits the expression of BUB1 gene in glioblastoma tumor cells, thereby reducing the proliferation and tumorigenicity of tumor cells *in vivo* and *in vitro* (Yu et al., 2019). Increased BUB1 expression signally facilitates cell proliferation, while decreased BUB1 expression restrains liver cancer cells proliferation (Zhu et al., 2020). The proliferation, migration, and invasion of PCa cell lines can be enhanced via BUB1B overexpression (Fu et al., 2016). Worse OS and DFS of HCC patients can be predicted by the high expression of BUB1B (Zhuang et al., 2018). CCNB1, an important protein regulating the G2/M (mitotic) cell cycle, is activated by Chk1, exerting its oncogenic role in colorectal cancer cells growth *in vivo* and *in vitro* (Fang et al., 2014). Abnormal FOXM1 expression can transcriptionally activate CCNB1 expression, thereby promoting the proliferation of HCC cells (Chai et al., 2018).

After exploring the potential information about the interactions between the seven hub genes and existing chemotherapeutic drugs, we found that various drugs could influence the expression levels of these hub genes. However, only topotecan, oxaliplatin and azathioprine could simultaneously reduce the expression level all seven hub genes. And it should be noted that further experiments are needed to support whether HCC patients with hub gene overexpression can benefit from hub gene inhibition or whether these key genes may be targets of drug treatment of tumor need ulteriorly biological experiments support.

In the current study, we have discussed that the development of HCC is associated with the overexpression of seven hub genes, which lead to poor overall survival, indicating that they may be considered as potential prognostic biomarkers for HCC. However, our study has several limitations: (1) some important clinical information (for example, different age, tumor size, TNM stage and grade) were not considered; (2) biological experiments must be carried out in the future to verify the results of our research; (3) the molecular mechanism of hub gene upregulation remains unclear. Therefore, the verification of hub genes will be the focus of our next work.

## Conclusion

Adopting a series of bioinformatics analysis methods, the current study identified 763 DEGs and seven hub genes (CDC20, CDK1, MAD2L1, BUB1, BUB1B, CCNB1, and CCNA2) that may be involved in hepatocellular carcinoma tumorigenesis and progression. Additionally, multiple database analysis and survival analysis demonstrated that these seven hub genes may regard as a latent prognostic biomarker and the overexpression of these seven hub genes might lead to reduced overall survival in HCC patients. These results provide a theoretical basis for the molecularly targeted therapy and prognosis evaluation of HCC.

## Data Availability Statement

The data in this study were obtained from the GEO (GSE121248) and TCGA public databases, and the acquisition and application methods complied with the corresponding database guidelines and policies.

## Author Contributions

ZL conceived the study, analyzed the raw data, and wrote the manuscript. YL prepared the diagrams in the manuscript. BC and QZ provided the useful suggestions in methodology. YC designed and instructed the research. All the authors have read and approved the final manuscript.

## Conflict of Interest

The authors declare that the research was conducted in the absence of any commercial or financial relationships that could be construed as a potential conflict of interest.

## Footnotes

1 https://www.ncbi.nlm.nih.gov/geo/

2 http://tcga-data.nci.nih.gov

3 https://david.ncifcrf.gov/

4 http://string-db.org/

5 http://www.cytoscape.org/

6 http://gepia.cancer-pku.cn/

7 https://www.oncomine.org/

8 https://www.proteinatlas.org/

9 http://ctdbase.org/

10 https://cytoscape.org/

## Supplementary Material

Supplementary Table 1 List of differentially expressed genes in GSE121248.

Supplementary Table 2 List of differentially expressed genes between HCC tissue and normal liver tissue in the TCGA dataset.

Supplementary Table 3 List of common differentially expressed genes both in GSE121248 and the TCGA-LIHC dataset.

Supplementary Table 4 Gene ontology enrichment analysis of the common differentially expressed genes both in GSE121248 and the TCGA-LIHC dataset.

Supplementary Table 5 Pathway enrichment analysis of the common DEGs both in GSE121248 and the TCGA-LIHC dataset.

Supplementary Table 6 STRING significance scores of common differentially expressed genes.
