# Supplementary material for: Identification and Analysis of Potential Key Genes Associated With Hepatocellular Carcinoma Based on Integrated Bioinformatics Methods
Source: Front Genet. 2021 Mar 9;12:571231. doi: 10.3389/fgene.2021.571231 (PMC7985067; doi:10.3389/fgene.2021.571231)
Supplement: Supplementary Table 3 — List of common differentially expressed genes both in GSE121248 and the TCGA-LIHC dataset. [file Table_3.DOCX]

x

1 HAMP

2 CXCL14

3 KCNN2

4 SPINK1

5 CNDP1

6 FCN2

7 TOP2A

8 CYP1A2

9 FCN3

10 THRSP

11 OIT3

12 GPC3

13 IDO2

14 CLEC4G

15 ASPM

16 COL15A1

17 APOF

18 TTC36

19 RRM2

20 ACSL4

21 BBOX1

22 HGFAC

23 CRHBP

24 SLC22A1

25 ANLN

26 CLRN3

27 AKR1B10

28 C7

29 GPM6A

30 DCN

31 CYP26A1

32 CTHRC1

33 LIFR

34 PBK

35 CXCL12

36 SRPX

37 CDKN3

38 NEK2

39 MT1M

40 LCAT

41 NAT2

42 C9

43 AKR1D1

44 SLC25A47

45 HGF

46 FOSB

47 CYP39A1

48 HMMR

49 GYS2

50 ESR1

51 HAO2

52 ECT2

53 CYP2B6

54 SRD5A2

55 LPA

56 DNASE1L3

57 GLYAT

58 SPP1

59 IGF2BP3

60 CDK1

61 PRC1

62 CCL20

63 DTL

64 BCHE

65 CDHR2

66 BUB1B

67 CCNB1

68 GLS2

69 NUF2

70 MT1F

71 BCO2

72 CAP2

73 SERPINE1

74 GHR

75 ALDOB

76 CYP4A11

77 DPT

78 CYP2A6

79 SULT1C2

80 ADH4

81 IL1RAP

82 EGR1

83 RACGAP1

84 ADH1B

85 CD5L

86 NCAPG

87 PGLYRP2

88 MAD2L1

89 TENM1

90 FOS

91 NUSAP1

92 GINS1

93 PRR11

94 PLAC8

95 CYP3A4

96 ZIC2

97 PDGFRA

98 CENPU

99 NDC80

100 MFSD2A

101 ROBO1

102 KIF20A

103 CCNB2

104 IGF1

105 ZG16

106 KMO

107 ENO3

108 MBL2

109 UHRF1

110 IGFALS

111 APOA5

112 ZWINT

113 TTK

114 SULT1E1

115 MOGAT2

116 RDH16

117 TACSTD2

118 CYP2E1

119 SORL1

120 HPD

121 ACSM3

122 TAT

123 IGFBP3

124 CYP2C8

125 EDIL3

126 UBE2T

127 RBM24

128 S100P

129 KIF4A

130 CYP2C9

131 CENPK

132 SDS

133 KDM8

134 DLGAP5

135 ATF5

136 KIF14

137 CETP

138 ADRA1A

139 HELLS

140 BIRC5

141 CENPF

142 FAM83D

143 BUB1

144 SPP2

145 AFM

146 PLG

147 PCK1

148 ANK3

149 HOGA1

150 CENPW

151 RAD51AP1

152 CYP2A7

153 CDC20

154 CFHR3

155 MT1G

156 ASPA

157 FAM13A

158 VNN1

159 ANXA10

160 KBTBD11

161 CTH

162 FBP1

163 TRPM8

164 EZH2

165 OLFML3

166 CYP2C19

167 CCBE1

168 F9

169 MME

170 PLCB1

171 GNMT

172 THBS1

173 STEAP4

174 LECT2

175 PTTG1

176 SLC3A1

177 NPY1R

178 NQO1

179 ADH1C

180 CYP8B1

181 MASP1

182 DIRAS3

183 ID1

184 ABCA8

185 HPGD

186 AVPR1A

187 PCDH9

188 KCND3

189 SQLE

190 GSTZ1

191 NR1I2

192 RND3

193 MARCO

194 E2F8

195 IL13RA2

196 GPD1

197 STAB2

198 CXCL2

199 CD109

200 LYZ

201 SMIM24

202 CFP

203 SLC10A1

204 SLC13A5

205 ITGA9

206 LHX2

207 KLKB1

208 SPATA18

209 C6

210 PBLD

211 GREM2

212 CPEB3

213 FCGR2B

214 HAL

215 ACOT12

216 XDH

217 ANGPTL6

218 ITGA2

219 NPC1L1

220 STXBP6

221 DEPDC1B

222 KPNA2

223 MELK

224 KIF11

225 OGDHL

226 FXYD1

227 LUM

228 MT1H

229 DNAJC6

230 FABP5

231 PLXNC1

232 CPS1

233 ADAMTS13

234 WDR72

235 CFHR4

236 COL4A1

237 ECM1

238 ALDH8A1

239 AADAT

240 KAZN

241 ALDH6A1

242 LCN2

243 APOBEC3B

244 EXPH5

245 ACADL

246 GAS2L3

247 ESM1

248 MASP2

249 SERPINA4

250 DKK1

251 COLEC11

252 RBMS3

253 C8A

254 MT1E

255 LYVE1

256 SOCS2

257 TFPI2

258 MRC1

259 ASS1

260 RCAN1

261 DNAJC12

262 CCDC34

263 EGR2

264 MAN1C1

265 MT1X

266 SLC22A7

267 AURKA

268 CCNE2

269 FLVCR1

270 ETNPPL

271 ADH6

272 PPP1R3B

273 SLC16A4

274 UBE2C

275 E2F7

276 SLC39A5

277 PZP

278 GPR158

279 C15orf48

280 HSP90AB1

281 MND1

282 RCL1

283 FAM149A

284 FTCD

285 PROZ

286 CSRNP1

287 CEP55

288 PTGS2

289 SLC27A5

290 PRG4

291 CYP3A43

292 MNS1

293 GCH1

294 POLE2

295 DEPDC1

296 ANO1

297 TBX15

298 GDPD1

299 MSH2

300 SFN

301 GJC1

302 MRO

303 LDHD

304 ST6GAL2

305 ANGPTL1

306 SLC17A2

307 KIF23

308 ADH1A

309 UBE2S

310 EPB41L4A

311 SAMD5

312 CDKN2C

313 HBB

314 SPC25

315 OTC

316 AKR7A3

317 CKAP2

318 EPHX2

319 GRAMD1C

320 FANCI

321 BHMT

322 DEPDC7

323 SLC7A2

324 CD1D

325 PPP1R1A

326 OIP5

327 SLC25A18

328 CCL19

329 KIF18B

330 DTNA

331 FANCD2

332 EPPK1

333 GMNN

334 INMT

335 STEAP3

336 IQGAP3

337 SOX6

338 NRCAM

339 VIPR1

340 CHML

341 RFC4

342 SERPINB9

343 RNF125

344 CDC7

345 TDO2

346 MCM8

347 OAT

348 ID4

349 BMPER

350 CCNA2

351 ENAH

352 ATOH8

353 PIGR

354 BGN

355 PDE11A

356 SULT2A1

357 ABI3BP

358 STMN1

359 CDCA3

360 CDKN2B

361 CDKN2A

362 C12orf75

363 PODXL

364 SLC26A2

365 CPN2

366 RGS5

367 FGFR2

368 PITPNM3

369 TMEM45A

370 IYD

371 GPT2

372 SIGIRR

373 ANXA2

374 TUBE1

375 FOXM1

376 MTFR2

377 EPHA2

378 SULF2

379 CPED1

380 BCAT1

381 SLC4A4

382 ITGA6

383 CYP4V2

384 DACH1

385 ACADS

386 LRRC1

387 EPB41L4B

388 TPPP2

389 SLC46A3

390 BDH2

391 CENPE

392 DPYS

393 MCC

394 STK39

395 TRIP13

396 CYP2C18

397 ADCY1

398 COLEC10

399 SMYD3

400 C11orf96

401 KIF2C

402 ACSL1

403 LGSN

404 AGXT2

405 CIDEB

406 HSD11B1

407 PDLIM5

408 CENPL

409 DMGDH

410 PON1

411 NRG1

412 CUX2

413 PLIN1

414 PTH1R

415 MCM4

416 TDRKH

417 ACACB

418 PHGDH

419 ZNF385B

420 TCTEX1D1

421 MROH2A

422 ACSM5

423 SLC27A2

424 PALM3

425 TP53I3

426 NEB

427 PHLDA1

428 GTSE1

429 ATAD2

430 LY6E

431 SERPINI1

432 CRISPLD2

433 MCM6

434 B3GNT5

435 SLC38A6

436 TSLP

437 ART4

438 PRSS8

439 FOXP2

440 SHBG

441 THY1

442 MKI67

443 HMGCS2

444 LEF1

445 RAB3B

446 FEN1

447 FETUB

448 CDCA7

449 SLC9B2

450 DIO1

451 CYP4F2

452 ACADSB

453 NR4A3

454 SOCS3

455 COL8A1

456 TPX2

457 PHYHD1

458 ETFDH

459 HMGB2

460 SHCBP1

461 SLC7A11

462 EHD3

463 EBF1

464 PPARGC1A

465 EDNRB

466 REEP6

467 TYMS

468 HSPB1

469 PANK1

470 LRRN3

471 CCL2

472 ZFPM2

473 FAM151A

474 SRD5A1

475 JDP2

476 FBLN5

477 CKS2

478 CDC37L1

479 F11

480 PTGIS

481 UNC93A

482 FABP1

483 HAO1

484 LPL

485 STIL

486 TOMM40L

487 SLC1A2

488 MS4A6A

489 RFX5

490 FAM110C

491 NR1I3

492 CDC25C

493 AGXT

494 LIPC

495 A1BG

496 MSRA

497 XPNPEP2

498 HSD17B2

499 NNMT

500 FEZ1

501 HOXA3

502 UGT3A1

503 DEFB1

504 PLCXD3

505 SLC17A3

506 C8B

507 SLC41A2

508 TREH

509 CDC6

510 PYROXD2

511 COL4A2

512 MT2A

513 LAPTM4B

514 MLIP

515 GADD45B

516 ZFP1

517 ANG

518 AR

519 PLVAP

520 SLC38A4

521 MCM2

522 NR4A2

523 ABCB4

524 ATF3

525 SFRP1

526 PCDH17

527 CD69

528 AKR1C3

529 TCF19

530 SLC25A15

531 TMCO3

532 ANKRD55

533 ADAMTSL2

534 ARHGEF26

535 G6PC

536 LOX

537 AZGP1

538 HRG

539 PLGLB2

540 HABP2

541 CLYBL

542 PIK3C2G

543 COL14A1

544 GAS1

545 DLG5

546 HOXA10

547 PCK2

548 LAMC1

549 GPR182

550 ACAA2

551 RELN

552 WDR76

553 TKT

554 ZBED8

555 GCKR

556 MAT1A

557 MDK

558 PBX1

559 IRAK1

560 KLF4

561 MYO10

562 FIGNL1

563 TTC39A

564 PDE7B

565 ASPN

566 TRIB1

567 SLC51A

568 MCM3

569 UROC1

570 FOXO1

571 IL1RL1

572 EPHA3

573 LMNB1

574 NR0B2

575 PPM1K

576 PRIM1

577 SLCO4C1

578 FNDC5

579 BRIP1

580 HSD17B6

581 FGF13

582 GSPT2

583 CKS1B

584 RNASEH2A

585 GCDH

586 N4BP2L1

587 ST3GAL6

588 COCH

589 TCF21

590 CENPH

591 CA5A

592 OLFML2B

593 HPX

594 TRIM16

595 S100A10

596 SERPINF2

597 GIPC2

598 SLC17A1

599 TBXA2R

600 ANKRD29

601 S100A8

602 DAO

603 RBP5

604 H2AFZ

605 UPB1

606 ARG1

607 OSBPL3

608 PSAT1

609 TBC1D31

610 SLC19A3

611 KCNMA1

612 MCM7

613 SERPINA7

614 TMEM154

615 SPATA6L

616 LAMA3

617 TRIM59

618 SMC4

619 GSTA1

620 SNRPB

621 SKAP1

622 ALDH1B1

623 CD200

624 CYP1A1

625 PRKAR2B

626 PIPOX

627 SYTL5

628 IRX3

629 RNF165

630 ADAMTSL3

631 ACSM2A

632 NAMPT

633 PAMR1

634 NTRK2

635 MECOM

636 PEG3

637 MXRA5

638 NGFR

639 TIGD1

640 FXYD2

641 TK1

642 AGMAT

643 AQP3

644 DPF3

645 CMBL

646 CA2

647 HPR

648 ZFP36

649 CAMK2B

650 SPDL1

651 GPR146

652 PKHD1

653 ACMSD

654 ACAA1

655 VASH2

656 GLDC

657 PSMD4

658 TM6SF2

659 STC1

660 GBP2

661 CNTN4

662 ABAT

663 GEM

664 PSPH

665 DCXR

666 C1R

667 SPRYD4

668 SLCO1B1

669 RBP7

670 SUCO

671 CDA

672 SLC28A1

673 RASGEF1B

674 SSR2

675 ABCA9

676 SPRY2

677 EGR3

678 LDLR

679 PTN

680 EXOC3L4

681 CDCA5

682 KNTC1

683 EBF2

684 IL33

685 AFP

686 MMAA

687 PLIN2

688 MOGAT1

689 EVA1A

690 EML6

691 ALAS1

692 DBH

693 SARDH

694 SPATS2

695 TBC1D16

696 SNRPE

697 NDRG2

698 EHHADH

699 GNA14

700 JUN

701 DHRS1

702 GNE

703 SEMA6D

704 CHEK1

705 SULT1A2

706 APOA1

707 CCDC3

708 ACTG2

709 SLC51B

710 SLC39A10

711 PLXDC1

712 CYP2J2

713 CENPJ

714 LONRF1

715 CP

716 TMEM100

717 SLC2A2

718 ABCG2

719 SLC6A12

720 GBP1

721 P2RY13

722 AGTR1

723 MCM5

724 HLF

725 TEK

726 DUSP5

727 TUBG1

728 GNAO1

729 RBP1

730 MCM10

731 NR3C2

732 FAHD2A

733 CD14

734 PLSCR4

735 TSPYL5

736 SEC14L2

737 MPPED1

738 AMDHD1

739 EPS8L3

740 IGSF3

741 PRODH2

742 AOX1

743 HS3ST3B1

744 CPT2

745 PLA2G5

746 DUSP6

747 KIF15

748 SIGLEC11

749 MTHFD1

750 USP2

751 HKDC1

752 IER2

753 ALDH2

754 FYN

755 EPHB1

756 BASP1

757 GDA

758 SULT1A1

759 PRKAG2

760 C1RL

761 APCS

762 LMNA

763 RBL1
